# Supplementary material for: Exploring the mediating effect of personality traits in the relationship between entrepreneurial intentions and academic performance among students
Source: PLoS One. 2023 Nov 8;18(11):e0293305. doi: 10.1371/journal.pone.0293305 (PMC10631684; doi:10.1371/journal.pone.0293305)
Supplement: S1 Dataset — (PDF) [file pone.0293305.s001.pdf]

Native	Age	Gender	Education	Course	Pocketmoney	Familymembers	Parentsoccupation	Parentsincome
2	4	2	6	1	1	2	2	3
3	3	1	5	1	1	2	2	4
3	3	2	5	1	1	2	1	3
3	3	2	6	1	2	1	2	4
3	3	1	6	1	4	2	2	3
3	3	2	6	1	1	2	1	4
3	3	2	5	1	2	2	2	5
3	3	2	6	1	1	2	3	1
3	3	1	5	1	2	1	2	1
3	2	1	5	1	1	2	2	3
3	3	1	5	1	1	2	2	2
3	3	1	6	1	2	2	2	3
3	3	1	6	1	1	1	3	2
3	3	1	6	1	1	2	2	3
3	3	2	6	1	1	2	3	2
2	5	1	6	1	4	1	4	1
2	4	2	5	1	1	2	2	2
4	3	1	6	2	3	1	1	3
2	4	1	6	2	2	2	2	3
2	3	1	6	2	2	1	2	2
1	3	2	6	2	3	1	2	4
3	3	1	6	2	1	2	3	1
3	2	1	4	4	2	2	2	5
3	2	1	4	4	2	1	1	3
3	2	1	4	4	5	2	3	3
4	2	1	4	4	1	2	2	2
1	2	1	4	4	2	2	2	5
3	2	1	4	4	1	1	2	4
3	2	1	4	4	1	2	1	2
3	2	1	4	4	1	2	2	2
3	2	1	4	4	1	2	1	4
3	2	2	4	4	1	2	1	5
3	2	1	5	4	1	2	3	4
5	2	1	4	4	1	2	2	3
3	3	1	5	4	1	2	3	1
3	3	1	5	4	1	2	2	3
3	3	1	4	4	1	2	3	1
3	2	1	5	4	1	2	3	1
3	2	1	5	4	1	2	3	1
4	2	1	4	4	1	2	2	3
5	3	2	4	4	3	2	3	5
3	2	1	4	4	1	2	1	5
4	3	1	4	4	1	1	1	5
3	2	1	4	4	1	1	1	5
3	2	1	4	4	2	2	2	5
4	2	1	4	4	1	2	2	5
2	2	1	4	4	2	2	1	5
4	2	1	4	4	1	2	1	4
1	3	1	4	4	1	2	2	4

4	2	1	4	4	1	2	3	4
2	3	1	4	4	1	2	1	5
4	3	1	4	4	1	1	1	5
4	2	1	4	4	1	2	3	4
4	2	2	4	4	2	2	3	5
6	2	1	4	4	2	1	2	5
4	2	2	4	4	1	2	2	1
3	2	2	4	4	2	2	1	5
1	3	2	4	4	1	2	3	5
3	2	2	4	4	2	2	1	3
4	3	1	4	4	2	2	2	5
3	2	1	4	4	1	2	3	3
2	3	1	4	4	2	2	3	4
3	2	1	4	4	1	2	1	2
3	2	1	4	4	1	1	3	2
2	2	1	4	4	2	2	2	5
1	2	1	4	4	1	2	3	4
3	2	1	4	4	1	2	2	2
4	2	1	4	4	1	3	2	1
4	2	1	4	4	1	3	3	3
3	2	1	4	4	1	2	2	4
3	2	1	4	4	2	2	2	5
4	2	1	4	4	1	2	2	4
2	2	1	5	4	2	2	3	3
3	2	1	4	3	1	2	3	1
3	2	1	4	3	1	2	2	4
2	2	2	5	3	1	2	2	5
2	3	1	4	3	2	1	1	2
3	2	1	4	3	1	1	2	4
2	2	1	4	3	1	1	2	4
2	2	1	4	3	2	2	2	5
2	2	1	4	3	1	2	3	1
4	2	1	4	3	1	1	2	5
3	2	1	4	3	1	3	2	5
6	2	1	4	3	2	2	3	5
4	2	1	4	3	2	2	3	2
1	3	2	6	3	1	1	3	2
1	4	2	6	3	1	1	1	1
3	3	2	5	3	1	1	2	1
4	4	2	6	3	4	2	2	5
3	4	1	6	3	1	1	2	5
1	2	1	4	3	2	2	1	5
3	2	1	5	3	1	2	2	2
3	2	1	5	3	1	2	1	3
3	2	1	4	3	1	1	2	1
3	2	1	4	3	1	2	2	1
3	2	1	4	3	1	2	2	1
3	2	1	5	3	1	2	3	1
3	2	1	5	3	1	2	1	1
3	2	1	5	3	1	1	3	1

3	2	1	4	3	1	1	2	1
3	2	1	4	3	1	2	3	1
3	2	1	5	3	1	2	2	3
3	2	1	5	3	1	2	1	2
3	2	2	4	3	1	2	3	1
4	5	5	4	5	5	5	4	5
4	5	5	4	5	4	5	4	5
4	5	5	4	4	4	4	4	5
5	5	5	4	4	5	4	4	5
4	4	5	4	4	5	5	5	5
4	5	5	4	5	4	5	4	5
4	5	5	4	5	4	5	4	5
4	5	5	4	5	4	5	4	5
4	4	5	4	5	5	4	5	4
4	5	5	5	4	5	4	4	5
4	5	5	4	5	4	5	4	5
4	5	5	4	5	5	4	4	5
4	4	5	4	5	4	4	4	5
4	5	5	4	5	4	5	4	5
5	4	5	4	4	4	4	5	2
4	4	5	4	4	5	4	4	5
5	4	4	5	4	4	4	4	5
4	5	5	4	5	4	5	4	5
5	4	4	4	5	5	4	4	5
4	5	5	4	5	4	5	4	5
4	5	5	4	5	4	5	4	5
4	4	5	4	5	5	4	4	5
4	5	5	4	5	4	5	4	5
4	5	4	4	5	4	5	4	5
5	5	5	5	4	4	4	5	5
4	5	5	4	4	5	5	4	5
4	5	5	4	5	4	5	4	5
4	5	5	4	5	5	4	4	4
4	4	1	1	1	1	3	4	2
4	4	4	5	4	4	5	4	5
4	5	5	4	5	4	5	4	5
4	5	5	4	5	4	5	4	5
4	5	4	5	5	4	5	4	5
4	4	5	4	5	5	4	4	5
5	4	5	5	5	4	5	5	5
5	5	4	4	4	4	4	4	4
5	5	5	4	5	5	4	5	5
5	3	3	3	3	3	4	3	4
5	5	5	5	5	5	5	5	5
4	5	4	4	4	4	5	4	4
3	3	3	3	3	3	3	4	4
4	5	5	4	4	4	4	4	5
3	3	3	3	3	3	3	4	3
4	5	4	5	4	5	4	5	4
4	4	4	4	4	4	4	4	4

5	5	5	5	5	5	5	5	5
4	4	3	4	3	3	4	4	4
4	1	4	4	4	3	4	4	4
4	4	2	4	3	3	4	4	3
4	4	3	5	4	2	5	4	4
5	5	5	5	5	5	5	5	5
5	5	2	5	2	2	5	5	5
4	4	3	2	2	2	3	4	3
3	4	3	4	3	3	3	4	4
3	4	5	5	4	3	3	3	3
5	5	1	5	5	1	5	5	1
3	3	3	3	3	4	4	4	3
3	3	3	3	4	3	4	3	3
5	5	2	5	3	4	4	4	4
3	4	2	3	4	2	4	3	3
4	4	4	4	4	4	4	4	4
5	5	2	4	3	4	4	3	3
4	2	3	3	2	1	4	1	4
4	4	5	4	4	4	4	4	5
2	3	1	5	4	2	1	3	3
4	4	5	5	5	5	5	4	5
4	2	4	1	4	5	1	4	1
1	2	5	1	2	4	5	2	1
5	4	5	5	4	5	5	4	5
1	2	2	4	1	1	5	1	4
5	4	5	4	4	5	5	5	4

Childrenin family	Siblingsstudying	Dependentsonparents	EIATE2	EIATE3	EIPBC4	EIPBC5	EIPBC6	EISN1
2	2	3	4	4	3	4	3	3
2	1	2	4	1	4	4	4	3
2	1	1	4	4	2	4	3	3
2	1	2	4	4	3	5	4	2
2	1	3	5	5	5	5	5	5
2	1	1	5	5	2	5	2	2
2	1	4	4	4	3	2	2	2
2	1	2	3	4	3	4	3	3
2	2	2	3	4	5	5	4	3
2	1	1	5	5	1	5	5	1
2	1	2	3	3	3	3	3	4
2	2	1	3	3	3	3	4	3
2	1	1	5	5	2	5	3	4
1	2	2	3	4	2	3	4	2
2	3	2	4	4	4	4	4	4
1	1	1	5	5	2	4	3	4
2	2	2	4	2	3	3	2	1
2	2	2	4	4	5	4	4	4
2	2	2	2	3	1	5	4	2
1	1	2	4	4	5	5	5	5
1	1	2	4	2	4	1	4	5
2	2	1	1	2	5	1	2	4
2	2	2	5	4	5	5	4	5
1	1	1	1	2	2	4	1	1
2	2	3	5	4	5	4	4	5
2	1	4	4	5	5	4	4	5
3	4	3	4	5	5	4	5	4
1	1	1	4	4	4	5	4	5
2	2	3	4	5	4	4	5	5
2	2	2	4	5	5	4	5	5
2	1	3	4	5	5	4	5	4
2	2	3	4	5	5	4	4	4
2	2	2	5	5	5	4	4	5
3	2	5	4	4	5	4	4	5
1	2	2	4	5	5	4	5	4
2	3	1	4	5	5	4	5	4
3	4	2	4	5	5	4	5	4
3	4	3	4	4	5	4	5	5
3	3	4	4	5	5	5	4	5
2	2	4	4	5	5	4	5	4
2	2	4	4	5	5	4	5	5
2	3	4	4	4	5	4	5	4
1	1	1	4	5	5	4	5	4
1	1	1	5	4	5	4	4	4
2	2	4	4	4	5	4	4	5
2	2	2	5	4	4	5	4	4
2	2	3	4	5	5	4	5	4
2	1	2	5	4	4	4	5	5
3	1	4	4	5	5	4	5	4

3	3	5	4	5	5	4	5	4
1	2	2	4	4	5	4	5	5
1	1	1	4	5	5	4	5	4
1	1	1	4	5	4	4	5	4
2	2	3	5	5	5	5	4	4
1	1	1	4	5	5	4	4	5
2	1	2	4	5	5	4	5	4
2	2	2	4	5	5	4	5	5
2	3	3	4	4	1	1	1	1
2	1	1	4	4	4	5	4	4
2	2	2	4	5	5	4	5	4
2	2	1	4	5	5	4	5	4
2	2	2	4	5	4	5	5	4
1	1	1	4	4	5	4	5	5
1	1	1	5	4	5	5	5	4
2	2	2	5	5	4	4	4	4
2	3	3	5	5	5	4	5	5
2	2	3	5	3	3	3	3	3
5	4	5	5	5	5	5	5	5
4	4	5	4	5	4	4	4	4
2	2	4	3	3	3	3	3	3
2	2	2	4	5	5	4	4	4
1	1	2	3	3	3	3	3	3
2	2	2	4	5	4	5	4	5
2	2	1	4	4	4	4	4	4
2	1	1	5	5	5	5	5	5
3	3	4	4	4	3	4	3	3
1	1	1	4	1	4	4	4	3
1	1	3	4	4	2	4	3	3
1	1	1	4	4	3	5	4	2
2	1	2	5	5	5	5	5	5
2	2	4	5	5	2	5	2	2
2	2	2	4	4	3	2	2	2
2	2	3	3	4	3	4	3	3
2	2	2	3	4	5	5	4	3
2	2	3	5	5	1	5	5	1
1	1	1	3	3	3	3	3	4
1	1	1	3	3	3	3	4	3
2	2	2	5	5	2	5	3	4
2	3	2	3	4	2	3	4	2
2	2	2	4	4	4	4	4	4
2	2	3	5	5	2	4	3	4
2	2	1	4	2	3	3	2	1
2	2	1	4	4	5	4	4	4
2	3	1	2	3	1	5	4	2
3	3	1	4	4	5	5	5	5
1	3	1	4	2	4	1	4	5
2	2	1	1	2	5	1	2	4
3	2	1	5	4	5	5	4	5
2	2	2	1	2	2	4	1	1

3	1	1	5	4	5	4	4	5
2	2	2	4	5	5	4	4	5
2	2	2	4	5	5	4	5	4
2	3	2	4	4	4	5	4	5
2	1	4	4	5	4	4	5	5
5	5	5	4	5	4	4	4	5
4	5	4	4	5	5	5	4	4
4	5	4	5	5	4	5	4	4
5	4	5	5	4	5	5	4	4
5	5	4	5	5	5	5	4	5
4	5	4	4	5	5	5	4	4
4	4	5	5	4	4	4	5	5
4	5	4	4	5	5	5	4	4
4	4	5	5	4	4	4	4	4
5	5	4	4	5	5	4	5	4
4	5	4	4	5	5	5	4	4
4	5	4	5	4	4	5	5	4
4	4	5	4	4	5	4	4	4
4	5	4	4	5	5	5	4	4
5	3	5	5	4	5	4	5	4
5	5	4	5	5	4	4	5	4
4	4	5	5	5	4	5	5	5
4	5	4	4	5	5	5	4	4
5	5	5	5	5	4	4	5	4
4	5	5	5	4	5	4	5	5
4	5	4	4	5	5	5	4	4
4	5	5	5	5	4	4	5	4
4	5	4	4	5	5	5	4	4
4	5	4	5	4	4	5	4	4
5	5	5	5	4	5	4	4	4
5	5	5	5	4	4	5	4	5
4	5	4	4	5	5	5	4	4
4	4	5	5	5	5	5	4	4
2	3	3	1	4	4	3	5	4
4	5	4	3	5	5	4	4	4
4	5	4	4	5	5	5	4	4
4	4	5	5	4	4	4	4	5
4	5	4	4	5	5	5	4	4
4	4	5	5	4	4	4	5	5
5	4	5	5	5	5	5	5	5
3	4	4	4	4	5	5	4	4
5	5	5	5	5	5	4	5	5
3	4	4	4	3	3	3	4	3
5	5	5	5	5	4	5	5	5
4	4	5	5	5	4	4	5	4
4	4	4	3	3	3	4	3	4
5	4	4	5	5	4	5	5	4
3	3	4	4	4	4	4	4	4
5	5	5	5	5	5	4	5	5
4	4	4	4	4	4	4	4	4

5	5	5	5	5	5	5	4	4
4	4	4	4	4	4	4	3	3
4	4	4	4	4	4	4	4	4
3	4	3	4	4	3	4	3	4
2	4	4	4	5	4	4	4	4
5	5	5	5	5	5	5	5	5
5	4	5	5	5	4	5	5	5
3	4	4	3	4	4	4	4	3
3	4	4	5	4	4	3	5	4
3	5	5	5	3	4	4	4	4
5	5	3	5	5	1	5	5	5
4	3	4	4	3	4	4	4	4
3	4	4	4	4	4	5	4	4
4	4	4	5	4	4	4	4	4
3	4	4	4	4	4	4	4	3
4	4	4	4	4	4	4	4	4
3	2	4	4	3	3	3	3	4
4	2	2	2	4	1	1	2	1
5	5	4	5	4	4	5	5	4
1	2	1	1	3	3	5	2	1
5	5	5	5	4	5	5	5	5
2	4	2	1	4	1	1	4	2
4	2	4	1	2	4	1	2	5
5	5	4	5	4	5	4	4	5
4	1	2	5	2	4	4	4	5
5	5	5	4	4	5	4	4	5

EISN4	EISN5	EIPES1	EIPES2	PTEP1	PTEP6	PTLOC2	PTLOC5	PTNFA3
4	4	4	4	4	4	4	4	4
4	4	4	4	4	4	4	4	4
4	4	3	3	4	3	4	4	3
5	4	4	2	4	4	4	5	4
5	5	5	5	5	5	5	5	5
5	5	5	5	4	5	5	5	4
3	4	3	3	4	4	3	4	4
3	4	4	3	4	4	5	4	4
3	3	3	3	5	5	5	3	4
5	5	1	5	5	3	5	5	1
4	4	3	4	3	4	4	3	4
4	3	3	3	4	4	4	4	4
4	4	4	4	4	4	5	4	4
4	3	3	3	4	4	4	4	4
4	4	4	4	4	4	4	4	4
4	3	3	3	2	4	4	3	3
4	1	4	4	2	2	2	4	1
4	4	5	5	5	4	5	4	4
1	3	3	1	2	1	1	3	3
5	4	5	5	5	5	5	4	5
1	4	1	2	4	2	1	4	1
5	2	1	4	2	4	1	2	4
5	4	5	5	5	4	5	4	5
5	1	4	4	1	2	5	2	4
5	5	4	5	5	5	4	4	5
4	3	4	5	4	5	5	4	4
5	4	5	4	4	5	5	4	4
4	5	4	5	5	4	4	4	5
5	4	5	5	4	5	5	4	4
5	4	5	5	5	5	4	5	4
5	4	5	4	5	4	4	5	5
4	4	5	4	5	4	5	5	4
4	4	5	5	4	5	5	4	5
5	5	5	5	5	4	5	5	5
5	4	5	4	5	4	4	5	5
5	4	5	4	4	5	5	4	4
5	4	5	4	5	4	4	5	5
4	5	4	4	4	5	5	4	4
4	4	5	5	5	4	4	5	5
5	4	5	4	5	4	4	5	5
4	4	5	4	5	4	5	4	4
4	4	5	4	4	5	4	4	5
5	4	5	4	5	4	4	5	5
4	5	2	5	3	5	5	4	5
4	4	5	5	5	4	5	5	4
4	4	5	4	4	5	5	5	4
5	4	5	4	5	4	4	5	5
4	4	5	5	5	5	5	5	4
5	4	5	4	5	5	5	4	5

5	4	5	4	5	4	4	5	5
4	4	5	4	5	5	5	5	4
5	4	5	4	5	4	4	5	5
5	4	5	4	5	4	5	4	4
4	5	5	5	5	5	5	4	5
5	4	5	5	5	5	5	4	4
5	4	5	4	5	4	4	5	5
4	4	4	4	4	5	5	5	5
3	4	2	2	3	3	1	4	4
5	4	5	4	5	4	3	5	5
5	4	5	4	5	4	4	5	5
5	4	5	4	4	5	5	4	4
5	4	5	4	5	4	4	5	5
4	4	5	4	4	5	5	4	4
5	5	5	5	4	5	5	5	5
4	4	4	3	4	4	4	4	5
4	5	5	5	5	5	5	5	5
4	3	4	3	4	4	4	3	3
5	5	5	5	5	5	5	5	4
5	4	4	4	4	5	5	5	4
3	4	4	4	4	4	3	3	3
4	4	5	5	4	4	5	5	4
3	4	3	3	3	4	4	4	4
4	5	4	5	5	5	5	5	5
4	4	4	4	4	4	4	4	4
5	5	5	5	5	5	5	5	5
4	4	4	4	4	4	4	4	4
4	4	4	4	4	4	4	4	4
4	4	3	3	4	3	4	4	3
5	4	4	2	4	4	4	5	4
5	5	5	5	5	5	5	5	5
5	5	5	5	4	5	5	5	4
3	4	3	3	4	4	3	4	4
3	4	4	3	4	4	5	4	4
3	3	3	3	5	5	5	3	4
5	5	1	5	5	3	5	5	1
4	4	3	4	3	4	4	3	4
4	3	3	3	4	4	4	4	4
4	4	4	4	4	4	5	4	4
4	3	3	3	4	4	4	4	4
4	4	4	4	4	4	4	4	4
4	3	3	3	2	4	4	3	3
4	1	4	4	2	2	2	4	1
4	4	5	5	5	4	5	4	4
1	3	3	1	2	1	1	3	3
5	4	5	5	5	5	5	4	5
1	4	1	2	4	2	1	4	1
5	2	1	4	2	4	1	2	4
5	4	5	5	5	4	5	4	5
5	1	4	4	1	2	5	2	4

5	5	4	5	5	5	4	4	5
4	3	4	5	4	5	5	4	4
5	4	5	4	4	5	5	4	4
4	5	4	5	5	4	4	4	5
5	4	5	5	4	5	5	4	4
5	4	5	4	4	4	5	5	4
5	4	4	4	5	4	5	4	4
5	5	5	5	4	5	5	5	5
5	5	5	4	5	4	5	5	5
5	4	5	5	5	4	4	5	4
5	4	4	4	5	4	5	5	5
4	5	4	4	5	4	5	4	4
5	4	4	4	5	5	4	5	5
5	4	4	5	4	5	5	5	5
5	4	5	5	4	5	4	5	4
5	4	4	4	4	5	4	5	5
5	4	4	4	4	5	4	5	4
5	5	5	4	5	4	5	5	5
5	4	4	4	5	4	5	4	4
5	4	4	3	5	4	4	4	5
4	4	5	5	5	5	4	5	4
4	4	5	4	4	4	5	5	4
5	4	4	4	5	4	5	4	4
4	5	5	4	4	4	5	5	4
4	4	4	5	4	4	5	4	5
5	4	4	4	5	4	5	4	4
5	4	5	4	4	5	4	4	5
5	4	4	4	5	4	5	4	4
5	5	5	5	5	5	5	5	5
5	5	4	5	4	5	4	5	4
4	4	5	4	5	5	4	5	5
5	4	4	4	5	4	5	4	4
5	4	5	5	4	5	4	5	5
5	3	4	4	4	3	1	2	1
4	5	5	5	4	4	4	5	4
5	4	4	4	5	4	5	4	4
5	4	5	5	4	5	4	5	4
5	4	4	4	5	4	5	4	4
5	4	4	4	5	5	4	5	4
5	5	5	5	4	5	5	4	5
5	5	5	4	5	5	5	5	5
5	5	4	5	5	5	5	5	5
3	3	4	4	4	4	4	4	4
5	5	5	4	5	5	5	5	5
4	5	3	4	4	4	4	4	5
4	4	4	4	4	4	4	4	4
4	4	5	5	4	4	4	4	4
4	4	4	4	4	4	4	4	4
4	5	4	4	5	4	5	4	5
4	4	4	4	4	4	4	4	4

5	5	5	5	5	5	5	5	5
4	4	3	4	3	4	4	4	4
4	3	4	4	4	3	4	4	3
4	4	4	4	4	4	4	4	3
4	5	4	4	4	4	4	4	4
5	5	5	5	5	5	5	5	5
5	5	5	4	5	5	5	2	4
4	4	4	4	4	4	4	4	4
3	4	4	3	5	5	5	5	4
5	4	2	4	4	5	5	5	3
5	5	5	5	4	5	5	5	5
4	4	2	3	4	4	4	4	4
4	3	4	4	4	4	4	4	4
4	4	4	5	4	4	4	4	4
3	3	4	4	3	3	3	3	3
4	4	4	4	4	4	4	4	4
5	5	2	2	4	4	4	4	4
4	1	2	2	2	2	4	2	4
4	4	5	4	5	4	4	5	4
4	2	2	1	1	1	2	3	1
5	5	4	5	5	5	4	5	5
2	1	2	2	3	1	2	4	2
1	2	1	1	2	4	5	1	1
4	5	5	5	5	4	5	4	5
4	5	4	4	4	4	5	4	5
5	5	4	4	5	4	5	5	4

PTRT4	PTRT2	PTEA6	NFA2	EA3	SAPSSE2	SAPSSE3	SAPSSG3	SAPSSG1
4	3	3	4	4	3	4	3	4
4	4	4	4	3	4	4	4	3
4	3	4	4	4	4	4	4	4
4	4	4	4	5	4	4	4	4
5	5	5	5	5	5	5	5	5
5	5	5	5	5	5	4	5	5
4	4	3	4	4	4	4	4	4
3	5	4	3	4	4	3	5	5
4	4	4	5	4	2	4	4	5
5	5	5	5	5	5	5	4	5
4	4	4	4	4	2	3	4	4
5	4	4	4	3	4	4	4	4
4	4	4	4	4	4	5	4	4
4	4	3	3	3	4	4	3	3
4	4	4	4	4	4	4	4	4
3	3	4	5	5	2	2	4	4
1	2	1	4	1	2	2	2	2
5	5	4	4	4	5	4	5	4
5	2	1	4	2	2	1	1	1
5	5	5	5	5	4	5	5	5
1	4	2	2	1	2	2	3	1
1	2	5	1	2	1	1	2	4
4	4	5	4	5	5	5	5	4
4	4	5	4	5	4	4	4	4
4	4	5	5	5	4	4	5	4
5	4	5	5	4	4	4	4	5
4	5	5	4	5	5	5	4	5
4	5	4	5	5	5	4	4	5
5	5	4	3	4	4	4	5	5
4	4	5	5	4	5	4	4	4
5	4	4	5	4	4	4	5	4
5	4	4	5	5	5	5	4	5
5	4	4	5	5	5	4	5	4
5	4	5	5	4	5	5	5	4
5	4	4	5	4	4	4	5	4
4	5	5	4	5	4	4	5	4
5	4	4	5	4	4	4	5	5
4	4	4	5	4	4	5	4	5
4	5	4	5	4	5	5	4	5
5	4	4	5	4	4	4	4	5
5	5	4	5	4	4	4	4	5
4	4	4	5	5	5	4	5	4
5	4	4	5	4	4	4	5	4
4	5	4	5	4	4	3	5	4
4	5	4	4	4	5	5	5	5
5	5	5	4	4	5	4	4	4
5	4	4	5	4	4	4	5	4
4	5	4	4	5	5	4	4	4
4	5	5	4	4	4	5	4	4

5	4	4	5	4	4	4	5	4
4	5	4	5	4	5	4	4	5
5	4	4	5	4	4	4	5	4
5	4	4	5	5	5	5	5	5
4	4	4	5	5	4	5	4	5
5	4	5	4	4	5	4	5	5
5	4	4	5	4	4	4	5	4
5	4	4	5	4	5	5	4	5
3	5	4	5	3	4	4	4	3
4	4	4	4	5	5	5	4	4
5	4	4	5	4	4	4	5	4
4	4	5	5	4	5	5	4	5
5	4	4	5	4	4	4	5	4
4	5	5	5	4	4	4	5	5
5	5	5	5	5	5	5	4	5
5	4	4	5	5	5	4	5	5
4	5	5	5	5	4	5	5	5
3	4	3	3	3	4	4	4	4
5	5	5	5	5	5	4	5	5
4	5	4	4	5	3	4	4	4
4	3	4	4	4	4	4	4	4
5	5	4	4	4	5	5	4	4
4	4	4	4	4	4	4	4	4
4	5	5	4	5	4	4	5	4
4	4	4	4	4	4	4	4	4
5	4	4	5	5	5	5	5	5
4	3	3	4	4	3	4	3	4
4	4	4	4	3	4	4	4	3
4	3	4	4	4	4	4	4	4
4	4	4	4	5	4	4	4	4
5	5	5	5	5	5	5	5	5
5	5	5	5	5	5	4	5	5
4	4	3	4	4	4	4	4	4
3	5	4	3	4	4	3	5	5
4	4	4	5	4	2	4	4	5
5	5	5	5	5	5	5	4	5
4	4	4	4	4	2	3	4	4
5	4	4	4	3	4	4	4	4
4	4	4	4	4	4	5	4	4
4	4	3	3	3	4	4	3	3
4	4	4	4	4	4	4	4	4
3	3	4	5	5	2	2	4	4
1	2	1	4	1	2	2	2	2
5	5	4	4	4	5	4	5	4
5	2	1	4	2	2	1	1	1
5	5	5	5	5	4	5	5	5
1	4	2	2	1	2	2	3	1
1	2	5	1	2	1	1	2	4
4	4	5	4	5	5	5	5	4
4	4	5	4	5	4	4	4	4

4	4	5	5	5	4	4	5	4
5	4	5	5	4	4	4	4	5
4	5	5	4	5	5	5	4	5
4	5	4	5	5	5	4	4	5
5	5	4	3	4	4	4	5	5
5	4	5						
5	4	5						
4	5	4						
4	5	5						
5	5	4						
4	5	4						
5	4	5						
4	5	5						
5	4	5						
5	5	4						
4	5	4						
5	4	4						
4	4	4						
5	4	5						
4	5	5						
4	5	4						
5	4	5						
5	4	5						
4	4	4						
4	4	5						
5	4	5						
5	5	5						
5	4	5						
4	5	5						
5	5	5						
5	4	4						
5	4	5						
5	5	4						
3	4	4						
5	5	4						
5	4	5						
5	4	4						
5	4	5						
4	5	4						
5	5	5						
5	5	5						
5	5	5						
4	4	4						
5	5	5						
3	3	4						
4	4	4						
5	4	4						
4	4	4						
5	4	5						
4	4	4						

5	5	5
4	4	4
3	4	3
3	4	4
4	4	4
5	5	5
4	5	5
4	4	4
4	3	5
3	3	3
5	5	5
3	3	2
4	4	3
4	4	4
3	3	3
4	4	4
3	3	3
2	2	4
4	4	4
2	1	2
5	5	5
1	1	1
2	1	2
4	4	5
5	5	4
5	4	5

SAPAC2	SAPAC1	SAPAB2	SAPAB4	SAPTM7	SAPTM1
4	4	4	4	4	4
4	4	3	3	4	3
4	4	3	3	4	4
4	4	4	4	4	4
5	5	5	5	5	5
5	2	4	4	5	5
4	4	4	4	4	4
5	5	4	4	3	5
5	5	3	3	3	3
5	5	5	5	5	5
4	4	4	3	3	2
4	4	4	4	4	3
4	4	4	4	4	4
3	3	3	3	3	3
4	4	4	4	4	4
4	4	4	3	3	3
4	2	4	2	2	4
4	5	4	4	4	4
2	3	1	2	1	2
4	5	5	5	5	5
2	4	2	1	1	1
5	1	1	2	1	2
5	4	5	4	4	5
5	4	5	5	5	4
5	5	4	5	4	5
4	4	4	5	5	4
4	5	5	4	5	4
4	5	5	4	5	4
4	4	5	4	4	5
5	5	4	5	4	5
5	4	4	5	4	5
5	5	5	4	5	4
5	5	5	4	5	5
4	5	4	5	5	4
5	5	5	4	5	4
5	4	4	5	4	5
4	5	5	4	5	5
5	5	5	5	4	5
4	5	4	5	5	4
4	5	5	4	5	4
4	5	4	5	4	4
5	5	5	4	4	4
5	4	4	5	4	5
4	4	5	4	5	5
4	5	4	4	5	4
5	5	4	5	4	5
5	4	4	5	4	5
5	5	4	4	4	4
5	4	5	4	4	5

5	4	4	5	4	5
4	4	5	5	5	5
5	4	4	5	4	5
5	5	5	4	5	5
4	5	4	5	5	5
4	5	5	5	4	4
5	4	4	5	4	5
4	5	5	5	5	4
1	2	1	3	4	4
4	5	4	5	5	4
5	4	4	5	4	5
4	5	4	5	4	4
5	4	4	5	4	5
4	5	4	4	5	4
5	4	5	5	5	5
5	5	5	5	5	5
5	5	5	5	5	5
4	4	4	4	4	4
5	5	5	5	5	5
4	4	5	3	3	4
4	4	4	4	4	4
4	4	4	5	4	4
4	4	4	4	4	4
5	4	5	5	4	5
4	4	4	4	4	4
5	5	5	5	5	5
4	4	4	4	4	4
4	4	3	3	4	3
4	4	3	3	4	4
4	4	4	4	4	4
5	5	5	5	5	5
5	2	4	4	5	5
4	4	4	4	4	4
5	5	4	4	3	5
5	5	3	3	3	3
5	5	5	5	5	5
4	4	4	3	3	2
4	4	4	4	4	3
4	4	4	4	4	4
3	3	3	3	3	3
4	4	4	4	4	4
4	4	4	3	3	3
4	2	4	2	2	4
4	5	4	4	4	4
2	3	1	2	1	2
4	5	5	5	5	5
2	4	2	1	1	1
5	1	1	2	1	2
5	4	5	4	4	5
5	4	5	5	5	4

5	5	4	5	4	5
4	4	4	5	5	4
4	5	5	4	5	4
4	5	5	4	5	4
4	4	5	4	4	5
