# Supplementary material for: Exploring the mediating effect of personality traits in the relationship between entrepreneurial intentions and academic performance among students
Source: PLoS One. 2023 Nov 8;18(11):e0293305. doi: 10.1371/journal.pone.0293305 (PMC10631684; doi:10.1371/journal.pone.0293305)
Supplement: Attachment — Submitted filename: Response to reviewer.docx [file pone.0293305.s002.docx]

**Response to Reviewer**

Subject: Response to Reviewer Comments - Manuscript ID [PONE-D-23-21632]

Dear Reviewer (s),

We sincerely appreciate your time and effort in reviewing our manuscript "Exploring the Mediating Effect of Personality Traits in the Relationship between Entrepreneurial Intentions and Academic Performance among Students." Your insightful feedback has been immensely valuable in refining the quality and rigor of our research.

We have carefully considered your comments and suggestions and made the necessary revisions to address them. The tracked changes have been highlighted in red. Below, we provide a detailed response to your comments:

| **SL** | **Comments (Reviewer 2)** | **Response** |
| --- | --- | --- |
| 1 | The introduction section would benefit from a few additional sentences to fortify its foundation. Additionally, elucidating the rationale behind introducing the concept of "entrepreneurial behavior" in the introduction, considering its absence from subsequent discussion and potential irrelevance to the study's context, is advisable. | We thank you for highlighting this point. In response to your suggestion, we have revised the introduction section to provide a more comprehensive elucidation of the rationale behind introducing the concept of "entrepreneurial behavior." The term has been rephrased without changing the context of the paragraphs. The concept of behaviors related to entrepreneurship is crucial in this study, even if it may not seem directly related. While the primary focus of our research is on the mediating effect of personality traits in the relationship between entrepreneurial intentions and academic performance, considering behaviors related to entrepreneurship adds depth and context to our research. Incorporating the concept of behaviors related to entrepreneurship within our study provides a broader theoretical framework. It helps create a more holistic understanding of the entrepreneurial ecosystem, allowing readers to connect the dots between intentions, behavior, and performance. Behaviors related to entrepreneurship are the tangible manifestation of entrepreneurial intentions. While our study may be centered on intentions and academic performance, understanding how intentions translate into actual behaviors can offer insights into the practical implications of our findings. |
| 2 | Within the literature review, the incorporation of more recent works, mainly spanning the years 2020 to 2023, would undoubtedly heighten the manuscript's value. | Your observation is indeed valuable. We have included a few recent works in the literature review section per your suggestion. And those works of the said period which were already cited have been highlighted in red. |
| 3 | In the discussion, succinctly encapsulating the novelty and significance of the primary discovery into a concise and groundbreaking assertion would be advantageous. | We appreciate your keen observation. Following your recommendation, we have revised the discussion section to succinctly encapsulate the pivotal discovery of our study. We have carefully crafted a concise statement that highlights the unique contribution of our research and emphasizes its potential implications for both academia and practical applications. |
| 4 | While the core arguments are lucidly expounded upon, specific paragraphs could benefit from refinement. Meticulous editing could significantly amplify the value of the content. Notably, instances of non-academic language and grammatical errors are discernible. Hence, a thorough editing pass is recommended by the author or a specialist to ensure consistent use of academic language and the effective conveyance of ideas. | In response to your valuable suggestion, we have thoroughly revisited the paragraphs and executed a comprehensive editing pass to enhance the quality and coherence of the content. We have diligently addressed instances of non-academic language and rectified grammatical errors to ensure a polished and professional presentation. |

We genuinely value your expertise and insights, which have undoubtedly contributed to the refinement of our manuscript. We are confident that our revisions have enhanced the manuscript's quality and coherence.

Thank you once again for your time and dedication to reviewing our work. We look forward to your evaluation of the revised manuscript.

Best regards,

Authors
